# Interpreting Gene Expression Effects of Disease-Associated Variants: A Lesson from *SNCA* rs356168

**DOI:** 10.3389/fgene.2017.00133

**Published:** 2017-09-20

**Authors:** Omolara-Chinue Glenn, Lidia Tagliafierro, Thomas G. Beach, Randy L. Woltjer, Ornit Chiba-Falek

**Affiliations:** ^1^Department of Neurology, Duke University Medical Center, Durham NC, United States; ^2^Center for Genomic and Computational Biology, Duke University Medical Center, Durham NC, United States; ^3^Banner Sun Health Research Institute, Sun City AZ, United States; ^4^Layton Aging and Alzheimer’s Disease Center, Department of Pathology, Oregon Health and Science University, Portland OR, United States

**Keywords:** SNCA gene, Parkinson’s disease, gene expression, translation of GWAS findings

## Abstract

The *SNCA* intronic single nucleotide polymorphism (SNP), rs356168, has been associated with Parkinson’s disease (PD) in large genome wide association studies (GWAS). Recently, the PD-risk allele, rs356168-G was shown to increase *SNCA*-mRNA expression using genome edited human induced pluripotent stem cells (iPSC)-derived neurons. In this study, as means of validation, we tested the effect of rs356168 on total *SNCA*-mRNA levels using brain tissues, temporal and frontal cortex, from healthy control donors. Carriers of the rs356168-G allele demonstrated a borderline significant decrease of *SNCA*-mRNA levels in temporal brain tissues (*p* = 0.02) compared to individuals homozygous for the ‘A’ allele. Similar trend, but weak, was observed in the analysis of frontal cortex samples, however, this analysis did not reach statistical significance. These results conflict with the recently reported effect of *SNCA* SNP rs356168 described above. Our study conveys the need to carefully interpret the precise molecular mechanism by which rs356168, or another tightly linked variant, affects the regulation of *SNCA* expression. The regulatory mechanisms that contribute to the observed associations between PD and the *SNCA*-3′ linkage disequilibrium region warrant further investigations.

## Introduction

Genetic associations of the *SNCA* gene have been reported with several neurodegenerative disorders that share the common pathology of Lewy bodies (LB), including familial and non-familial Parkinson’s disease (PD) (Pals et al., 2004; Mueller et al., 2005; Maraganore et al., 2006; Mizuta et al., 2006; Ross et al., 2007; Winkler et al., 2007; Myhre et al., 2008; Pankratz et al., 2009; Satake et al., 2009; Simon-Sanchez et al., 2009, 2011; Edwards et al., 2010; Mata et al., 2010; Spencer et al., 2011). *SNCA* missense mutations and multiplications were identified in few families with autosomal dominant form of PD (Polymeropoulos et al., 1997; Singleton et al., 2003). However, the precise genetic variants within the *SNCA* gene that contribute to non-Mendelian PD and related synucleinopathies, and their molecular mechanisms of action, are largely unknown. In the post genome-wide association studies (GWAS) era, we are shifting gears toward translation of genetic disease loci to molecular mechanisms of pathogenesis and pinpointing the causal genetic factors and their functional effects. Various technologies and approaches including *in vitro* and *in vivo* model systems are being developed continuously to advance this field of inquiry. One of these approaches is eQTL analysis using tissues relevant to the studied disease.

Single nucleotide polymorphism (SNP) rs356168, positioned in intron 4 of the *SNCA* locus and tagging the *SNCA*-3′ linkage disequilibrium (LD) region, is among the top ranked PD-associated SNPs (Nalls et al., 2014). Specifically, the rs356168-A allele was reported to exert a protective effect in a large scale meta-analysis with an odds ratio (OR) of 0.79 (95% CI, 0.76–0.81) and *p* = 2.70*e*-50 (Nalls et al., 2014). Recently, using the innovative CRISPR/Cas9 genome editing technology in human pluripotent stem cells (iPSCs), Soldner et al. (2016) reported that the PD-risk allele, rs356168-G caused increased *SNCA*-mRNA expression. They further showed a SNP-dependent binding of transcription factors (TFs) EMX2 and NKX6-1 and proposed that the effect of SNP rs356168 on *SNCA-*mRNA levels and PD risk is mediated by an enhancer regulation of transcription via interaction with these TFs (Soldner et al., 2016).

Here, to validate the reported findings and establish their relevance to PD mechanism, we performed an *in vivo* study to assess whether rs356168 regulatory effect on *SNCA* expression is present in aging human brains by analyzing human brain tissues from aged unaffected donors from whom high-quality *post-mortem* tissues were available.

## Materials and Methods

### Study Samples

The study cohort consisted of neurologically healthy individuals (*N* = 134). The unaffected brain samples were obtained from *post-mortem* tissues of clinically normal subjects who were examined, in most instances, within 1 year of death and were found to have no cognitive disorder or parkinsonism and neuropathological findings insufficient for diagnosing PD, Alzheimer’s disease (AD), or other neurodegenerative disorders. All donors were whites and unrelated. Demographics and neuropathology for these subjects are summarized in **Table 1**. The project was approved by the Duke Institution Review Board (IRB). The methods were carried out in accordance with the relevant guidelines and regulations.

**Table 1:** Demographic description.

Characteristic	Normal
Total no.	134
White, %	100
Male, %	59.8
Age at death, mean ± SEM	80.5 ± 1.1
PMI (hr), mean ± SEM	9.6 ± 0.9
Rs356168-A, %	55.2


*^∗^Rs356168-A frequency in EUR population is 51.7% (dbSNP147, 1006 chromosomes submitted by 1000genome).*

All frozen brain tissues, frontal cortex (FC, *N* = 127) and temporal cortex (TC, *N* = 106), were obtained from rapid autopsy through the Kathleen Price Bryan Brain Bank (KPBBB) at Duke University, the Banner Sun Health Research Institute Brain and Body Donation Program (Beach et al., 2015) and the Layton Aging and Alzheimer’s Disease Center at Oregon Health and Science University (Supplementary Table 1).

### SNP Genotyping

Genotype determination of the SNP was performed by allelic discrimination using TaqMan SNP Genotyping Assays and carried out using the ABI ViiA7, following the manufacturer’s protocol (Applied Biosystems, Foster City, CA, United States). All genotypes were tested for Hardy–Weinberg Equilibrium.

### RNA Extraction and cDNA Synthesis

Total RNA was extracted from brain samples (100 mg) using TRIzol reagent (Invitrogen, Carlsbad, CA, United States) followed by purification with an RNeasy kit (Qiagen, Valencia, CA, United States), following the manufacturer’s protocol. RNA concentration was determined spectrophotometrically and the quality of sample and lack of significant degradation was confirmed utilizing an Agilent Bioanalyzer. The RNA Integrity Number (RIN) measurements were greater than seven, validating the RNA quality control. Next, cDNA was synthesized using MultiScribe Reverse Transcriptase (RT) enzyme (Applied Biosystems, Foster City, CA, United States), following the manufacturer’s protocol.

### Real-Time PCR

Real-time PCR was used to quantify the levels of human *SNCA*-mRNA (Chiba-Falek et al., 2005, 2006; Cronin et al., 2009; Linnertz et al., 2009). Briefly, duplicates of each sample were assayed by relative quantitative real-time PCR using the ABI ViiA7 to determine the level of *SNCA* message (ID Hs00240906_m1, 62bp, best coverage for the different *SNCA*-mRNA isoforms) in brain tissues relative to the geometric mean of mRNAs encoding the human neuronal proteins Enolase 2 (*ENO2*, ID Hs00157360_m1, 77bp) and Synaptophysin (*SYP*, ID Hs00300531_m1, 63bp) (Applied Biosystems, Foster City, CA, United States). Expression fold differences were calculated as 2^-ΔΔCt^ (Livak and Schmittgen, 2001); ΔCt = [Ct = [Ct (*SNCA*) - Ct (*reference*)]. ΔΔCt = [ΔCt (*sample*)] - [ΔCt (*calibrator*)].

### Statistical Analysis

All analyses were carried out using SAS statistical software, Version 9.3 (SAS Institute, Cary, NC, United States). Expression levels of *SNCA* mRNA of each sample were measured in replicate and the results were averaged. The mean expression of a group of samples was reported as mean ± SE. We assessed the associations of the expression traits (*SNCA-*mRNA) with SNP-rs356168 genotypes using the Generalized Linear Model procedure (PROC GLM). A log transformation (log2) was performed on all mRNA levels to assure normal distributions (Bengtsson et al., 2005). For each brain region (TC and FC), we performed two statistical models. An additive genetic model was used and genotypes were coded with 0, 1 or 2 copies of the ‘A’ allele. A dominant genetic model was used whereas G was defined as the dominant allele. All models included sex, age, tissue source and Post-Mortem Interval (PMI) as covariates. Correction for multiple testing (correction factor 2 for two brain regions) employed the Bonferroni method.

## Results

### The Effect of SNP rs356168 on *SNCA*-mRNA Levels

The analyses were performed using *post-mortem* matched brain tissues from unaffected individuals to directly assess the genetic contribution to the regulation of *SNCA* expression, avoiding other confounding factors arising from the neurodegeneration associated with PD. A total of 134 individuals were included in the analysis, for 104 individuals both temporal and frontal cortex tissues were available for the study. Genotypes of SNP rs356168 (G/A) were determined and allele frequency for the entire cohort was calculated (**Table 1**).

First, we assessed the associations with confounding factors that might affect RNA levels. No significant associations of total *SNCA*-mRNA levels were observed in temporal (TC, *N* = 106) and frontal cortex (FC, *N* = 127) with sex, age, RIN, or PMI. Tissue source was marginally associated with the *SNCA*-mRNA expression levels in temporal cortex (*P* < 0.05) but not in frontal cortex. All of the subsequent analyses included tissue source, sex, age, RIN, and PMI as covariates.

**FIGURE 1:**
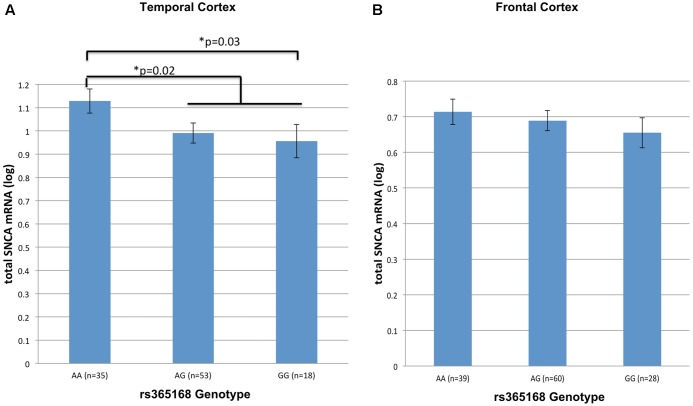
The effect of rs356168 on *SNCA* mRNA expression levels in human brain tissues. The study cohort consisted of unaffected control brain tissues from Caucasian donors. The subjects were genotyped for rs356168. Fold levels of human *SNCA*-mRNA were assayed by real-time RT-PCR using TaqMan technology, and calculated relative to the geometric mean of *SYP-* and *ENO2-* mRNAs reference control, using the 2^-ΔΔ*C*_T_^ method (i.e., results presented are relative to a specific brain RNA sample). The values presented here are means levels ± SE adjusted for age, sex, tissue source and PMI. GLM analysis was used to test the association of rs356168 with *SNCA* mRNA expression levels in the **(A)** temporal cortex (TC), and **(B)** frontal cortex (FC). Significant differences are denoted by ^∗^.

Next, we tested the effect of rs356168 on *SNCA*-mRNA levels using two regions of brain tissues, temporal and frontal obtained from healthy control (*N*_TC,FC_ = 106, 127) donors. In the temporal cortex, we detected a suggested dose effect of rs356168 on *SNCA* mRNA levels that showed a trend toward significance (*P* = 0.07) (**Figure 1A**). The homozygous rs356168 ‘protective’ AA genotype (*n* = 35) showed significantly higher expression levels of *SNCA*-mRNA in the temporal cortex than the combined GA and GG genotypes (*n* = 53 and 18, respectively), amounting to nearly 17% increase (*p* = 0.02, **Figure 1A** and Supplementary Table 2). This effect remained significant post-multiple testing correction for two brain regions (*p* = 0.04).

The same trends and direction, although weak, of allele-dose effect was observed in the analysis of matched frontal cortex samples from healthy control group. Homozygous AA (*n* = 39) showed increased levels of *SNCA*-mRNA compared to the carriers of the G allele, heterozygous GA (*n* = 60) and the homozygous GG (*n* = 28) (**Figure 1B** and Supplementary Table 2). However, the expression effect was relatively very small (∼5%) and didn’t reach significance (*p* = 0.4).

Overall, genotypes containing the rs356168-G allele were associated with decrease *SNCA*-mRNA expression in the temporal cortex tissues. In addition, we performed a comparison analysis between the homozygous groups, the mean of *SNCA*-mRNA levels was ∼20% lower in individuals homozygous for the G allele (GG, *n* = 18) relative to individuals harboring the AA genotype (*n* = 35) (*p* = 0.03, **Figure 1A** and Supplementary Table 2). Similarly, in the frontal cortex GG individuals demonstrated a modest, ∼9%, decrease in the mean of *SNCA*-mRNA levels compared to AA individuals (*p* = 0.22, **Figure 1B** and Supplementary Table 2). However, these expression differences between the homozygous genotypes were statically significant only in the temporal cortex and after correction for multiple comparisons (two analyzed brain tissues) showed only borderline significance (*p* = 0.06).

## Discussion

It has been suggested that the regulation of *SNCA* expression levels are critical for the development of PD (Tagliafierro and Chiba-Falek, 2016). Here, we studied the association of a PD-associated SNP with total *SNCA*-mRNA levels using an assay with best coverage for *SNCA* transcript forms. We analyzed unaffected brains, which allow us to overcome methodological and interpretative challenges that arise from the massive cell loss, particularly neuronal loss, along with other pathologic processes accompanying neurodegeneration that may influence expression. We demonstrated that individual carriers of the PD-risk allele rs356168-G exhibited decreased *SNCA*-mRNA levels in the temporal cortex compared to homozygous of the PD-‘protective’ allele (‘AA’). Expression Quantitative Trait Loci (eQTL) analysis using the available GTeX datasets showed a similar trend (AA > GG) for several human brain regions, but except from suggestive association for the cerebellar hemisphere (*p* = 0.055) these associations were not statistically significant possibly due to the small sample sizes (Supplementary Figure 1 and Table 3). However, these results conflict with the reported effect using the iPSC-derived model (Soldner et al., 2016). It is possible that difficulties in quantifying the total *SNCA* transcripts levels affected the validity of the reported conclusions. In fact, while our quantification assay reflected on the total levels of *SNCA* transcripts, we noticed that the allele-specific assay used to measure *SNCA*-mRNA was designed to target only the long 3′UTR isoform of *SNCA* transcript and therefore the method Soldner et al. (2016) used to quantify *SNCA*-mRNA levels did not capture all *SNCA* transcript species. It is crucial to note that the long 3′UTR isoform of *SNCA* is not as abundant as the short 3′UTR isoform and represents only a small fraction of *SNCA* transcripts [TargetScan 7.0 (Agarwal et al., 2015)]. Furthermore, Soldner et al. (2016) also used quantitative RT-PCR to measure total *SNCA*-mRNA in frontal cortex from a comparable sample size to our study, however, combined both healthy and PD subjects. Our results of the frontal cortex analysis did not replicate their findings; while they reported a significant increase in total *SNCA*-mRNA in carriers of the G allele (*p* = 0.037) (Soldner et al., 2016), we did not detect a significant effect of SNP rs356168 on *SNCA*-mRNA levels in frontal cortex of unaffected individuals. Nevertheless, the non-significant modest trend we observed in the frontal cortex mirrors our findings in the temporal cortex. It is possible that these contradicting results reflect the technical limitations of eQTL analysis in *post-mortem* brain tissue, particularly analysis using neurodegenerative affected brain samples (Dumitriu et al., 2012). Alternatively, methodological differences such as different assay probes used for best coverage of *SNCA* isoforms (exons boundary 3–4 vs. 5–6), and different reference gene/s used for normalization (geometric mean of *SYP-* and *ENO2* vs. *GAPDH* only) could also explain the inconsistent observations. In addition, sample size is another limitation that can possibly explain the contradictory results. This is also exemplified in our analysis of the dataset available through GTeX. The GTeX eQTL data demonstrated the effect of the AA genotype on higher *SNCA* expression in several brain regions, however, it showed no effect of rs356168 on *SNCA* expression in other brain regions, and in some opposing trends (Supplementary Figure 1). The GTeX cohort available for the analysis of the rs356168 effect on *SNCA* expression was small at the brain region level hence these results could be artifacts of limited power (Supplementary Table 3). Furthermore, our analysis of the frontal cortex showed only a small effect on *SNCA* expression, thus, our sample size may be under-power to detect significant association with small expression differences. Therefore, robust replication studies are needed using larger independent cohorts.

Now, in the post genome-wide association (GWA) era the fundamental question is, which are the actual causal variants within disease-associated genomic regions and what are their mechanisms of action. The genome edited iPSC-derived system is a powerful tool to model the functional consequences of neurodegenerative disease-associated non-coding variants and represents a strong *in vitro* system to follow-up on GWAS discoveries. However, other non-genetic factors such as, aging presumably modify epigenetic states and can influence gene regulation (Qiang et al., 2013; Doege and Abeliovich, 2014). These events of potential relevance to neurodegenerative diseases in aging are “erased” in iPSC-derived models that mimic fetal/juvenile neurons unless they undergo particular protocols to induce aging (Miller et al., 2013). Therefore, one needs to interpret the reported results of the PD-risk SNP rs356168 with caution. Different approaches, such as expression of progerin in iPSC-derived neurons (Miller et al., 2013), and direct conversion into induced Neurons (iNs) (Mertens et al., 2015; Huh et al., 2016), have been established to differentiate neurons that retain aging-related signatures including epigenetic state and transcriptomic profile. Further investigation of the *cis*-genetic effect on the regulation of *SNCA* expression using homogenous population of mature and aged iPSC-derived neurons, or alternatively iNs, is warranted. As a general remark, the replication of *cis*-regulatory effects using different complementary strategies, including the genome edited iPSC-derived system, will demonstrate the robustness of the results and provide strong support for a putative functional role of the studied candidate disease variant/s.

Rs356168 was one of the top reported SNPs associated with PD risk (Nalls et al., 2014). Chromatin state segmentation (chromHMM track) using the Roadmap Epigenomics data for the brain temporal and frontal lobes, and substantia nigra, annotate the region (∼1 kb) that contains rs356168 as an active enhancer. Nevertheless, one couldn’t exclude the possibility that this SNP serves as a marker for the actual causal variant that is in high LD and possibly within this enhancer segment. Noteworthy, it has been suggested recently that the focus on SNPs misses much of the genetic variation (Huddleston and Eichler, 2016), and at the same time there has been increased support for the idea that short structural variants (SSVs) may have a large impact on many human complex traits and gene expression variations (Pearson et al., 2005; Mirkin, 2007; Willems et al., 2014; Sudmant et al., 2015; Gymrek et al., 2016; Huddleston and Eichler, 2016; Saul et al., 2016) and that haplotypes matter (Gross et al., 2017). Thus, deep systematic assessment of the *SNCA* 3′-LD block using long-read sequencing technologies is required to decipher the genetic variants underlying the PD-GWAS signals.

It is widely agreed that up regulation of *SNCA*-mRNA contribute to disease risk (Tagliafierro and Chiba-Falek, 2016). We found here that the ‘protective’ allele of rs356168 is associated with higher *SNCA*-mRNA levels. Interestingly, the same trend was observed previously with other SNPs that tag the *SNCA* 3′ region using a different cohort of unaffected brain tissues (Linnertz et al., 2009). Although these findings run contrary to the conventional hypothesis that higher *SNCA* expression confers PD-risk, it might be that SNPs in the *SNCA* 3′ LD block exert their regulatory effect not simply by changing total *SNCA*-mRNA levels, at least not solely, but also by other molecular mechanism/s of gene expression regulation. Previous reports suggested that PD-associated SNPs in the *SNCA* 3′ also affect pre-mRNA processing mechanisms, such as splicing regulation of the proceeding exon 5 resulting in *SNCA*112 isoform (Lee et al., 2002; Beyer, 2006; McCarthy et al., 2011), and/or selection of alternative polyadenylation site resulting in the longer 3′UTR isoform (Rhinn et al., 2012). In fact, as discussed above, the effect of the PD-risk allele rs356168-G that Soldner et al. (2016) observed was actually on the increased generation of the longer 3′UTR isoform. Presumably, these alternative mechanisms of gene regulation may promote the generation of putative “pathogenic” isoform/s encoded by *SNCA*, and/or efficiency of translation. In-depth follow up investigations of these alternative molecular mechanisms in the context of PD-pathogenesis will shed light on the development of genetic biomarkers and potential therapeutic targets.

## Author Contributions

OC-F: Conceived aims, conceptual design and strategy design, data analysis, interpretation of the results, wrote and approved manuscript. O-CG: Performed experiments, generation of the genetic data, data analysis, wrote and approved manuscript. LT: Data analysis, interpretation of the results, wrote and approved manuscript. TB and RW: Generation of reagents, provided clinical and pathological data, wrote and approved the manuscript.

## Conflict of Interest Statement

The authors declare that the research was conducted in the absence of any commercial or financial relationships that could be construed as a potential conflict of interest. The reviewer UD and handling Editor declared their shared affiliation.
