# Supplementary material for: Interpreting Gene Expression Effects of Disease-Associated Variants: A Lesson from SNCA rs356168
Source: Front Genet. 2017 Sep 20;8:133. doi: 10.3389/fgene.2017.00133 (PMC5611418; doi:10.3389/fgene.2017.00133)
Supplement: Supplementary file 1 [file Table_1.DOCX]

| Source | Normal Subjects | PMI, mean ± S.E.M. |
| --- | --- | --- |
| Duke ADRC | 56 | 10.95 ± 1.09 |
| Oregon Health Science | 29 | 14.51 ± 2.17 |
| Maryland | 2 | 23.00 ± 0.71 |
| Banner Sun Health | 47 | 4.40 ± 1.46 |
| All Sources, total | **134** | **9.60 ± 0.91** |

**Supplementary Table 1.** PMI by Brain Bank resource
