# Supplementary material for: Interpreting Gene Expression Effects of Disease-Associated Variants: A Lesson from SNCA rs356168
Source: Front Genet. 2017 Sep 20;8:133. doi: 10.3389/fgene.2017.00133 (PMC5611418; doi:10.3389/fgene.2017.00133)
Supplement: Supplementary file 2 [file Table_2.DOCX]

**Supplementary Table 2.** Total *SNCA*-mRNA relative levels (log)

| **Temporal Cortex** | **(TC)** |  |
| --- | --- | --- |
| Genotype | Mean | St.Dev. |
| AA (n=35) | 1.12883399 | 0.05206663 |
| AG (n=53) | 0.99081434 | 0.04312982 |
| GG (n=18) | 0.95605196 | 0.07161132 |
|  |  |  |
| **Frontal Cortex** | **(FC)** |  |
| Genotype | Mean | St.Dev. |
| AA (n=39) | 0.71399745 | 0.03563037 |
| AG (n=60) | 0.68906397 | 0.02848748 |
| GG (n=28) | 0.65486968 | 0.04204674 |
