# Supplementary material for: Interpreting Gene Expression Effects of Disease-Associated Variants: A Lesson from SNCA rs356168
Source: Front Genet. 2017 Sep 20;8:133. doi: 10.3389/fgene.2017.00133 (PMC5611418; doi:10.3389/fgene.2017.00133)
Supplement: Supplementary file 3 [file Table_3.pdf]

Supplementary Table 3

Human Brain Region	Sample Size	P---Value	Supp Figure 1 panel
Anterior_cingulate_cortex_BA24	72	0.11	1A
Caudate_basal_ganglia	100	0.41	1B
Cerebellar_Hemisphere	89	0.055 (AA>AG,GG)	1C
Cerebellum	103	0.23	1D
Cortex	96	0.58	1E
Frontal_Cortex_BA9	92	0.69	1F
Hippocampus	81	0.51	1G
Hypothalamus	81	0.24	1H
Nucleus_accumbens_basal_ganglia	93	0.76	1I
Putamen_basal_ganglia	82	0.96	1J

The Genotype-Tissue Expression (GTEx) Project was supported by the Common Fund of the Office of the Director of the NaYonal InsYtute of Health, and by NCI, NHGRI, NHLBI, NIDA, NIMH, and NINDS. The data used for the reported analyses were obtained from the GTEx Portal on 05/02/17.
