# Supplementary material for: Interpreting Gene Expression Effects of Disease-Associated Variants: A Lesson from SNCA rs356168
Source: Front Genet. 2017 Sep 20;8:133. doi: 10.3389/fgene.2017.00133 (PMC5611418; doi:10.3389/fgene.2017.00133)
Supplement: Supplementary file 4 [file Image_1.pdf]

Supplementary Figure 1A

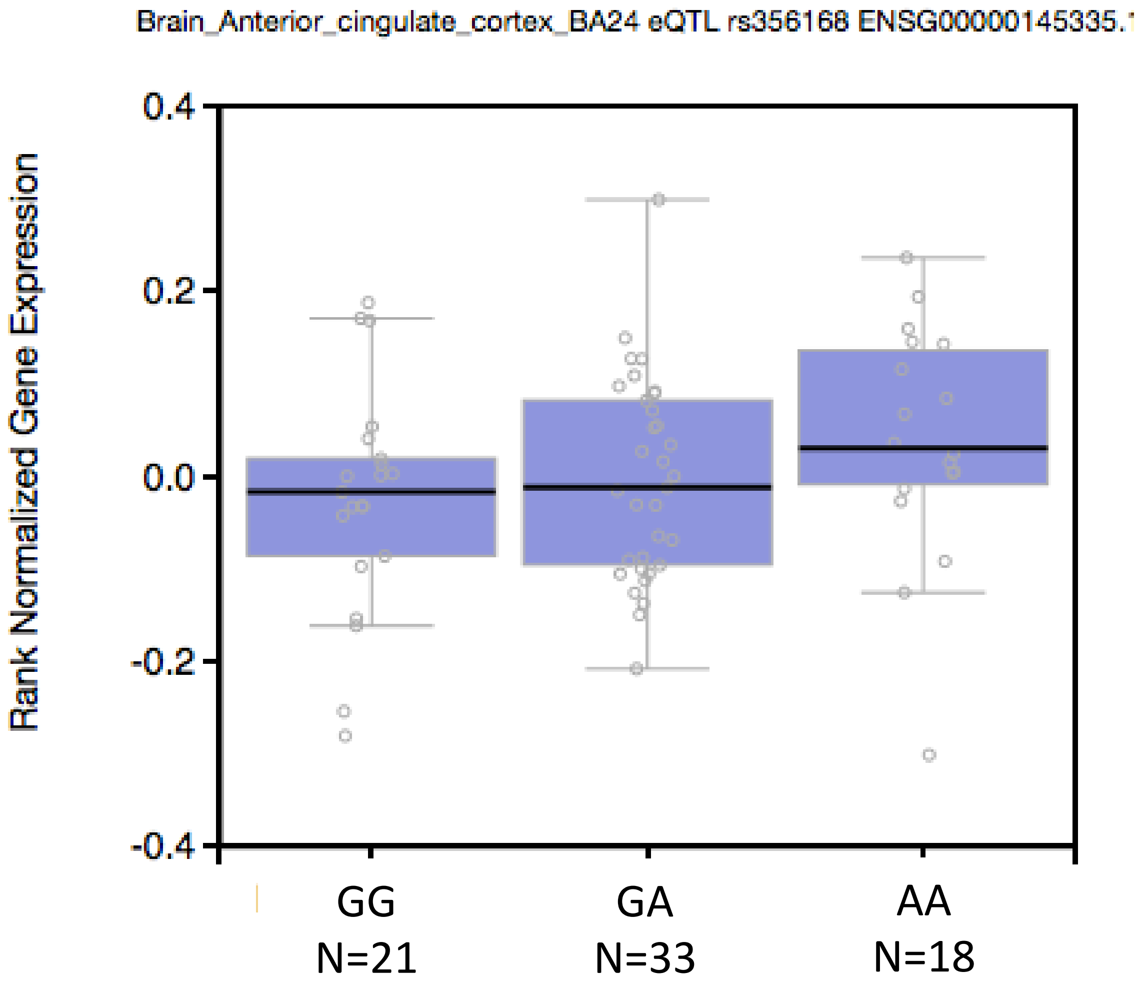

Supplementary Figure 1B

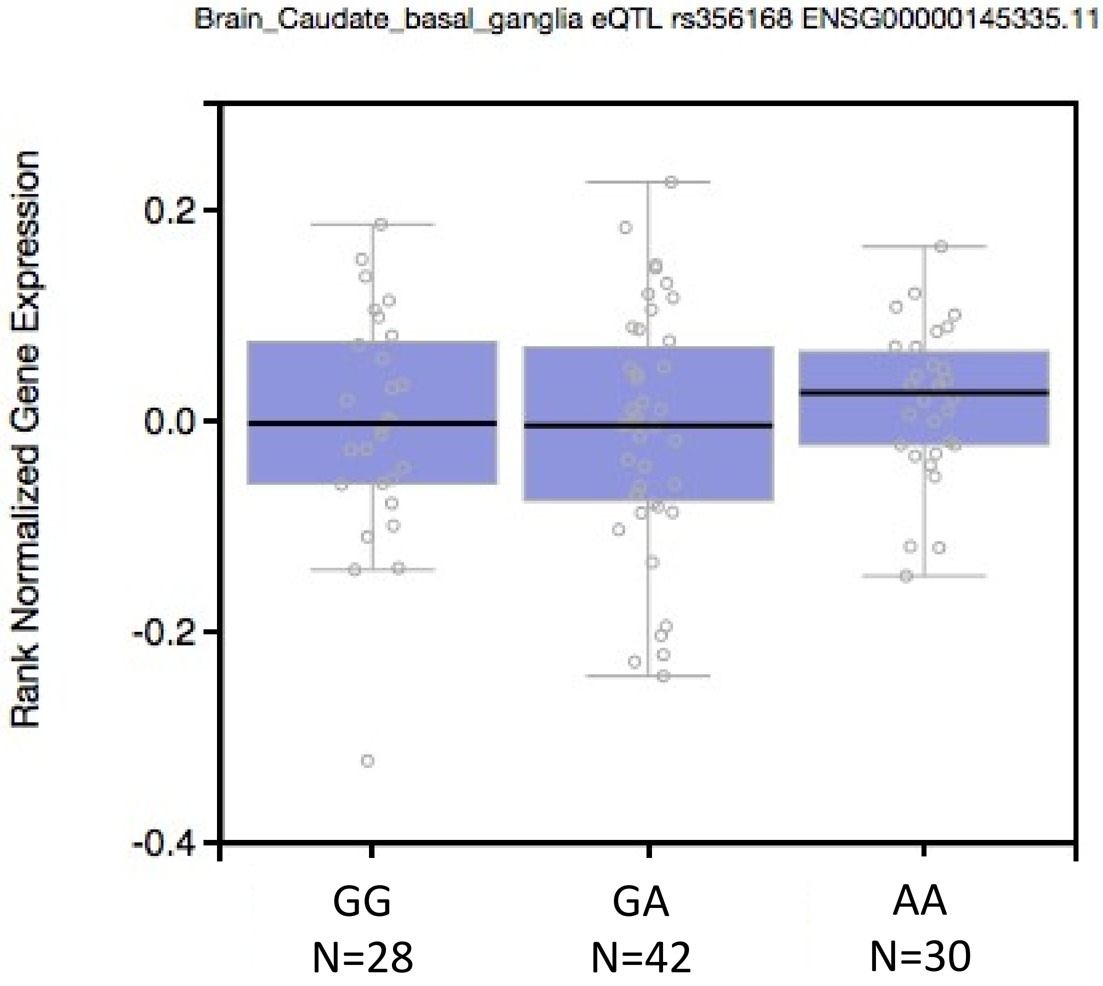

Supplementary Figure 1C

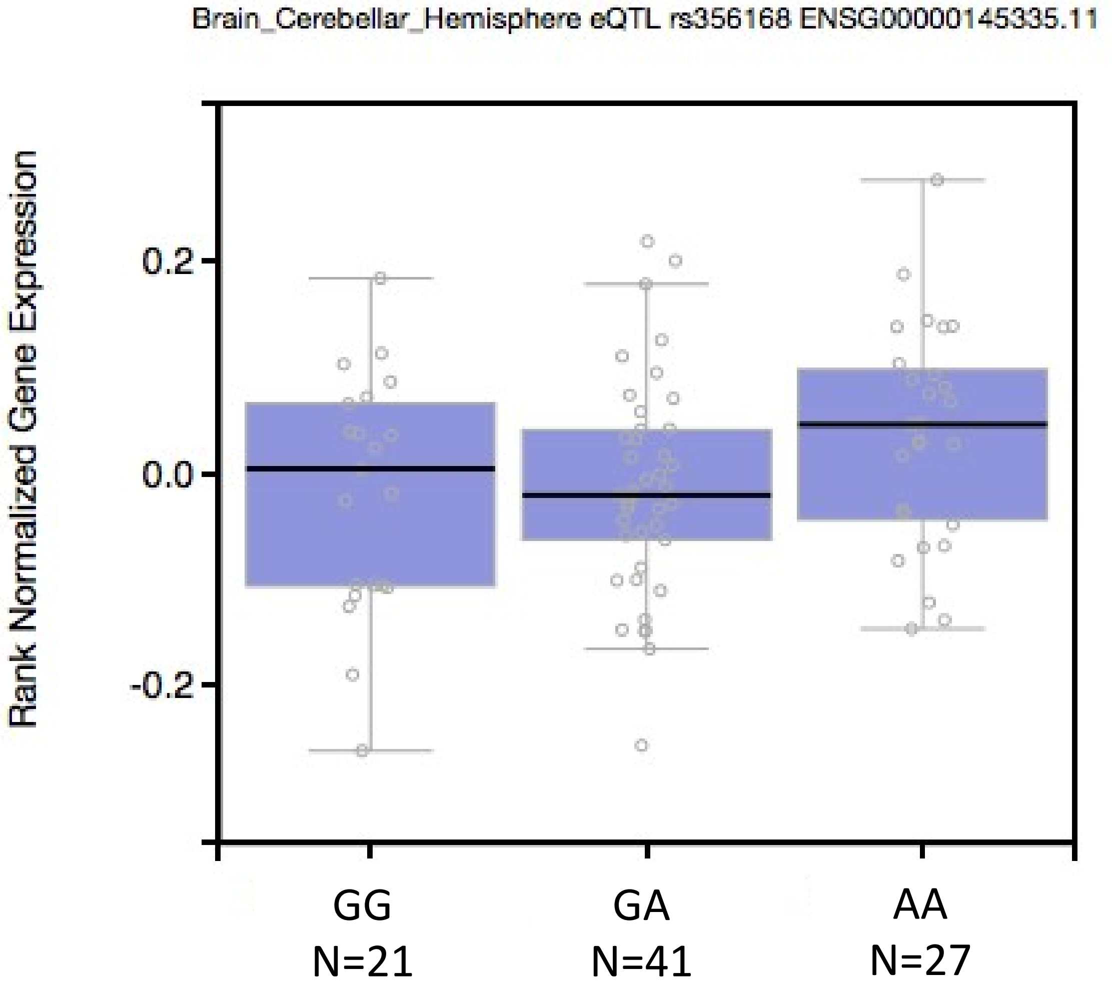

Supplementary Figure 1D

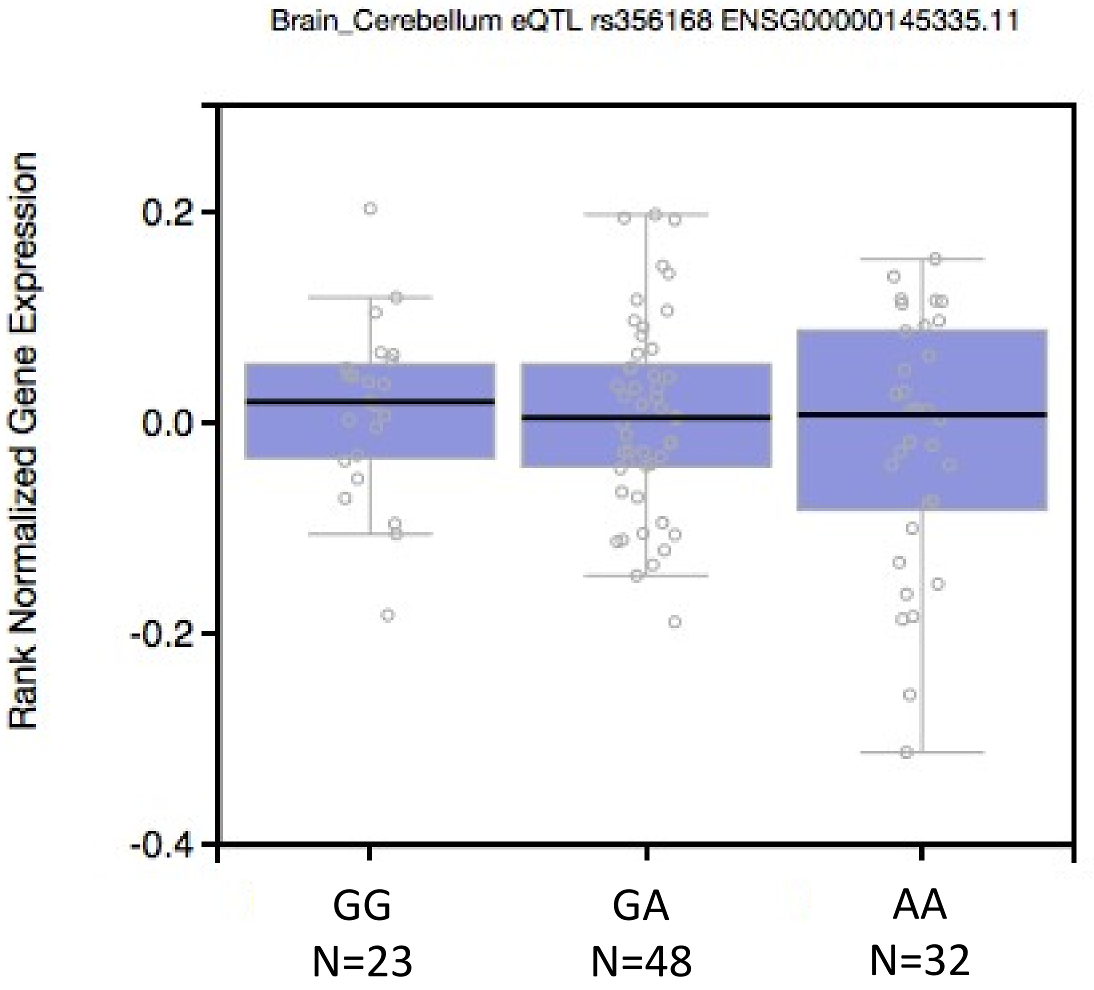

Supplementary Figure 1E

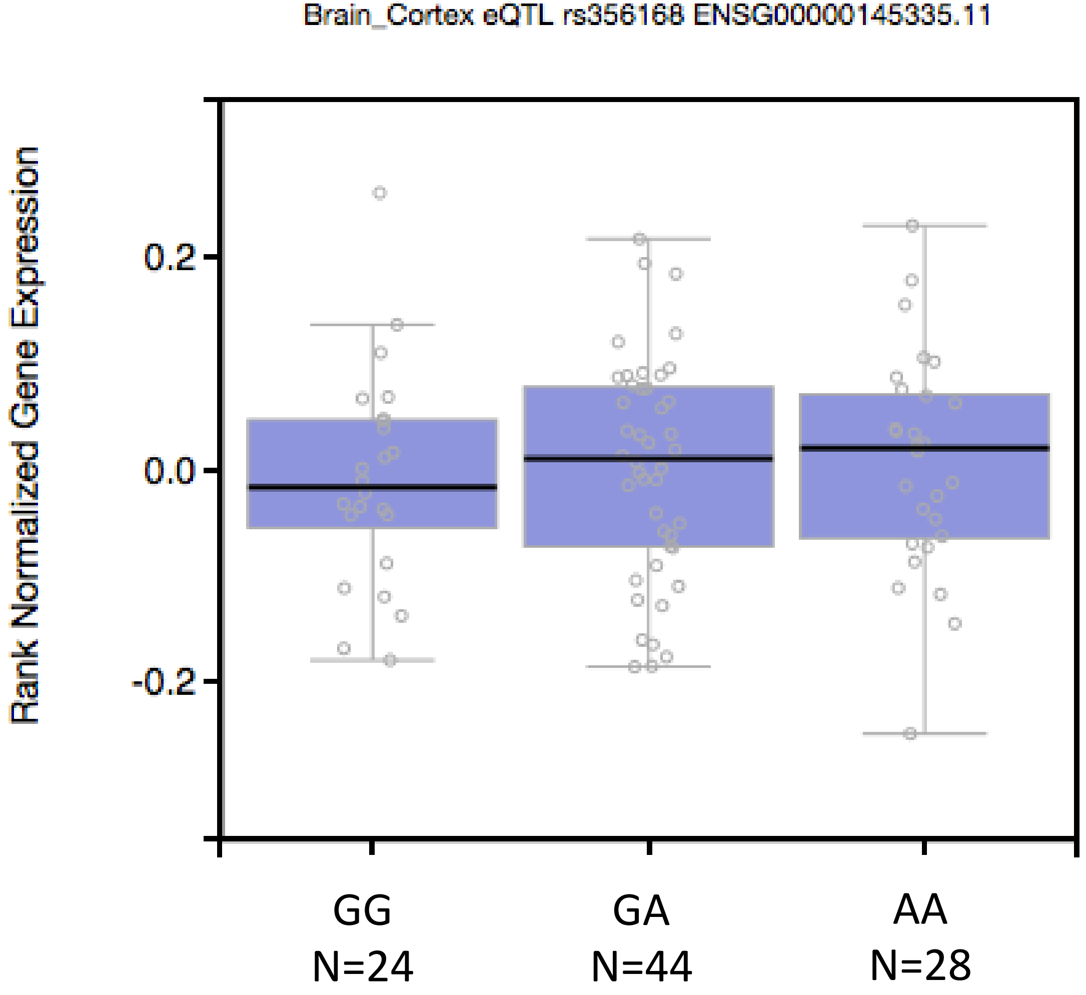

Supplementary Figure 1F

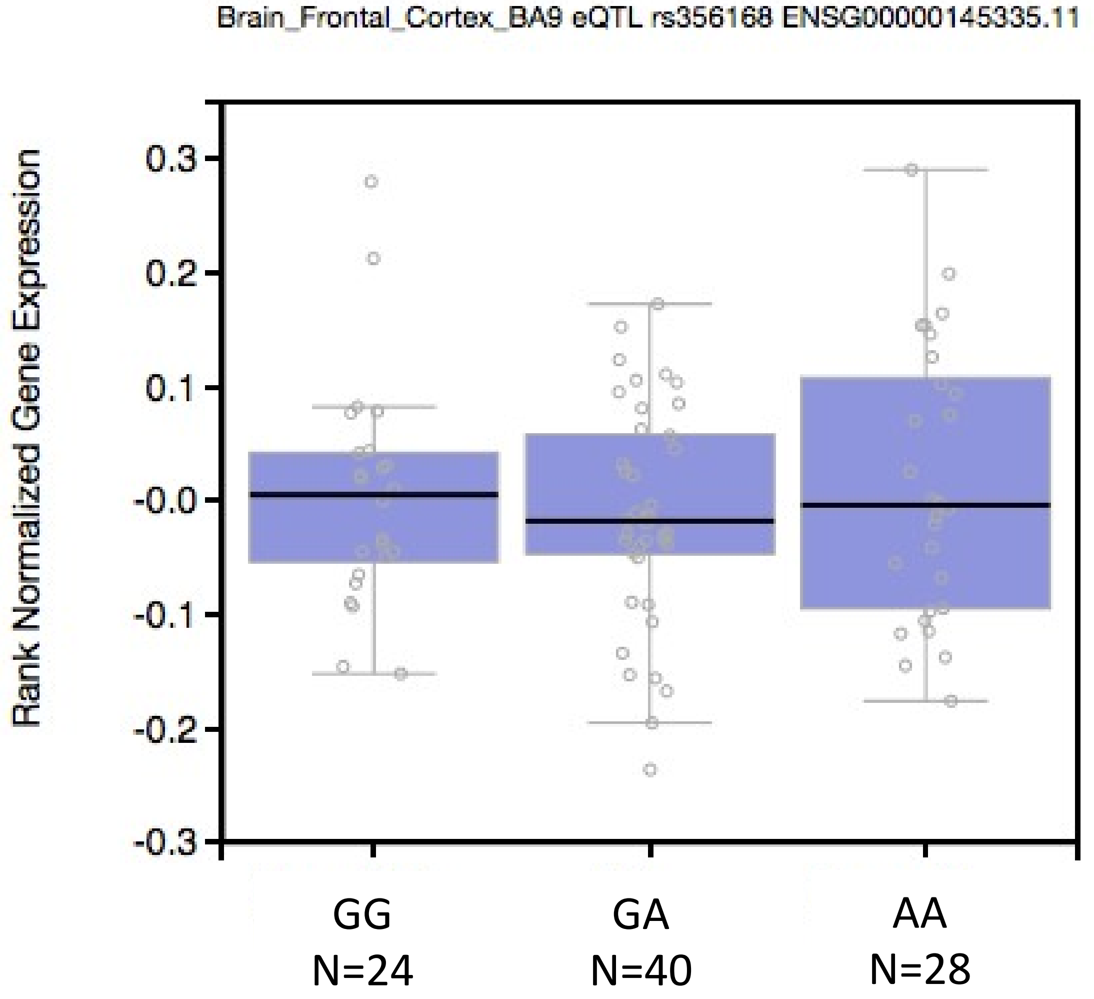

Supplementary Figure 1G

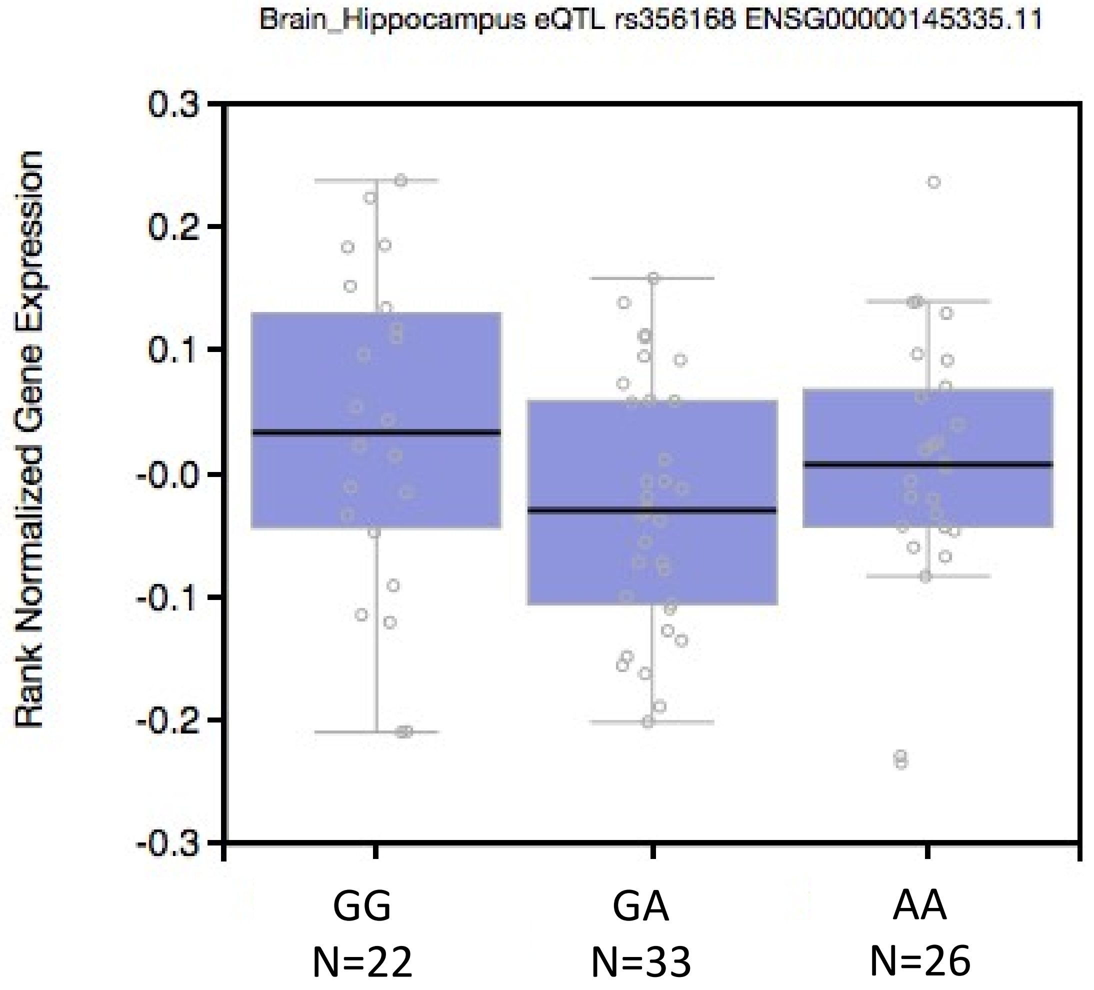

Supplementary Figure 1H

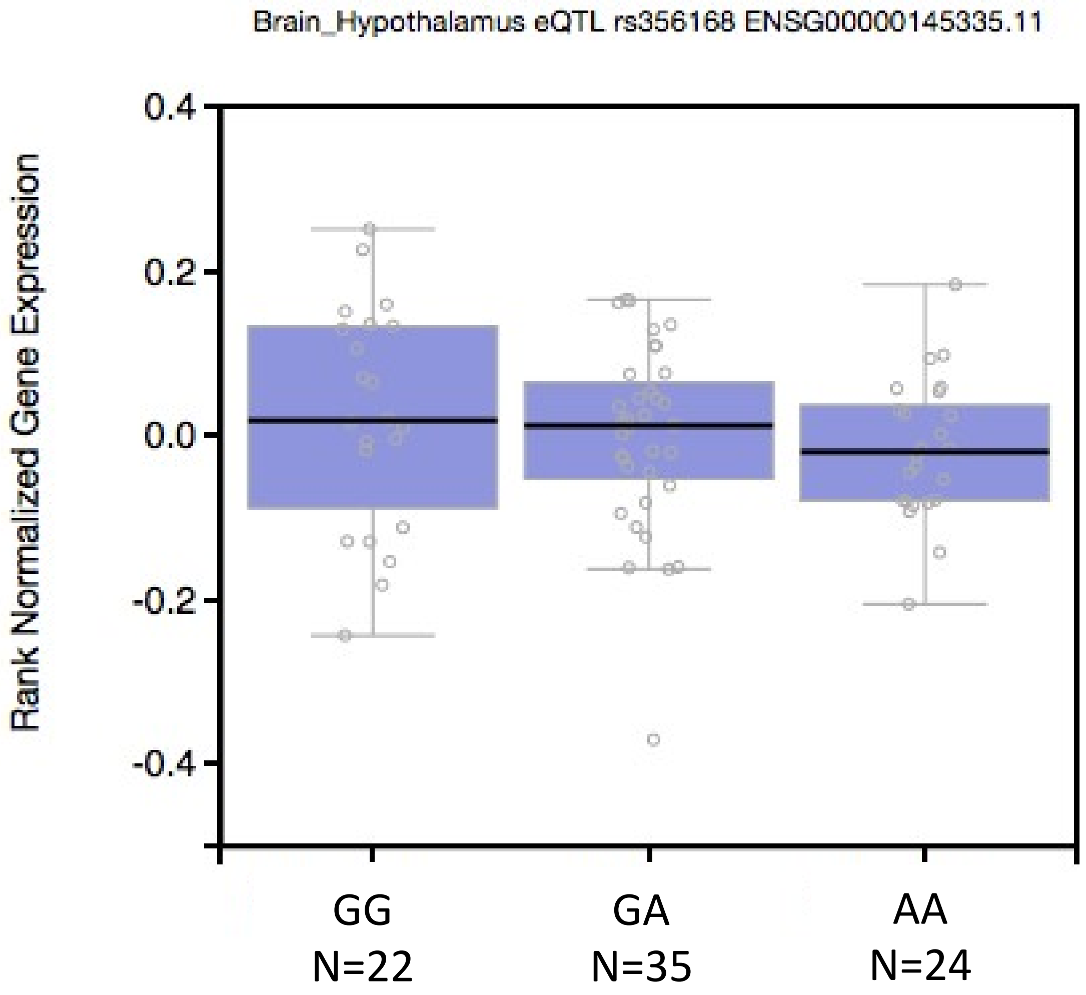

Supplementary Figure 1I

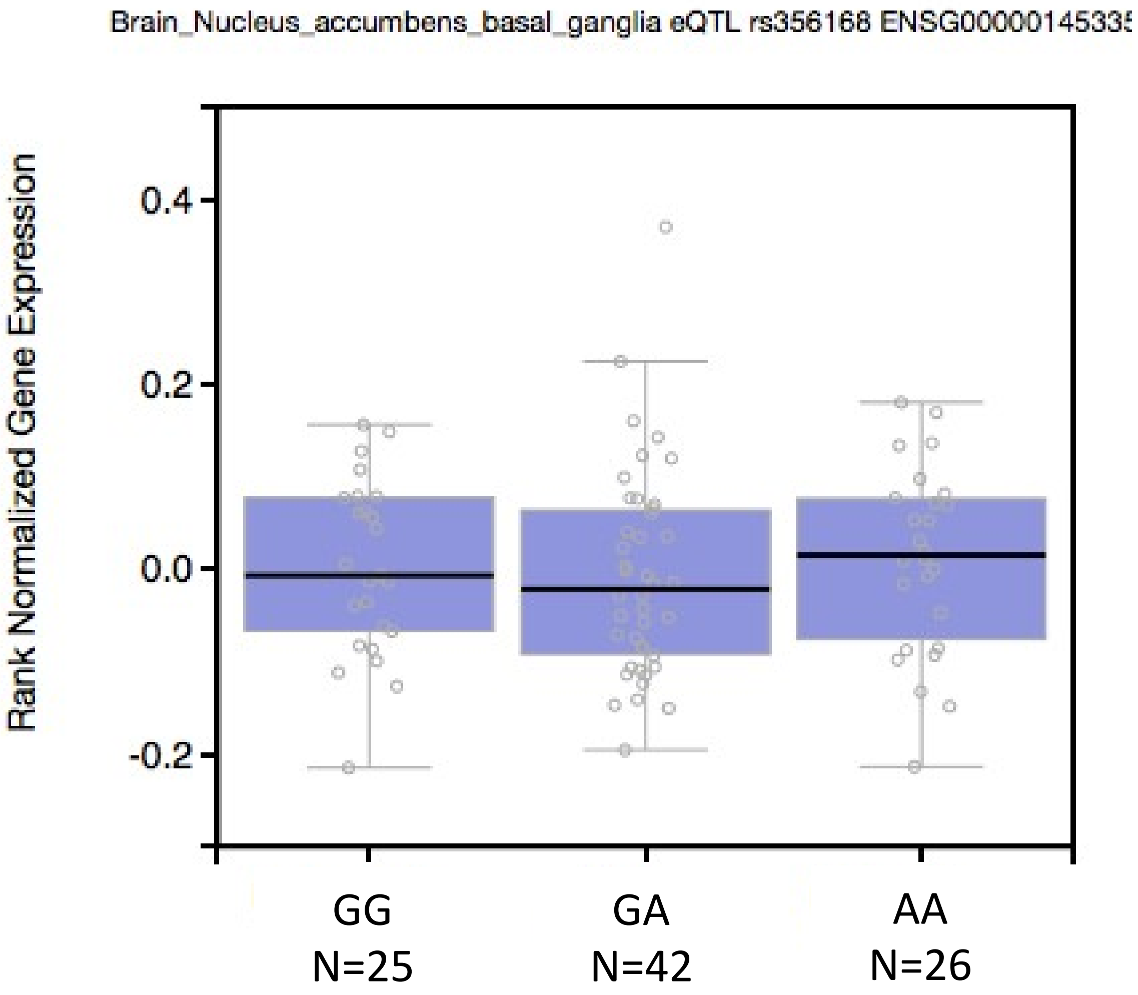

Supplementary Figure 1J

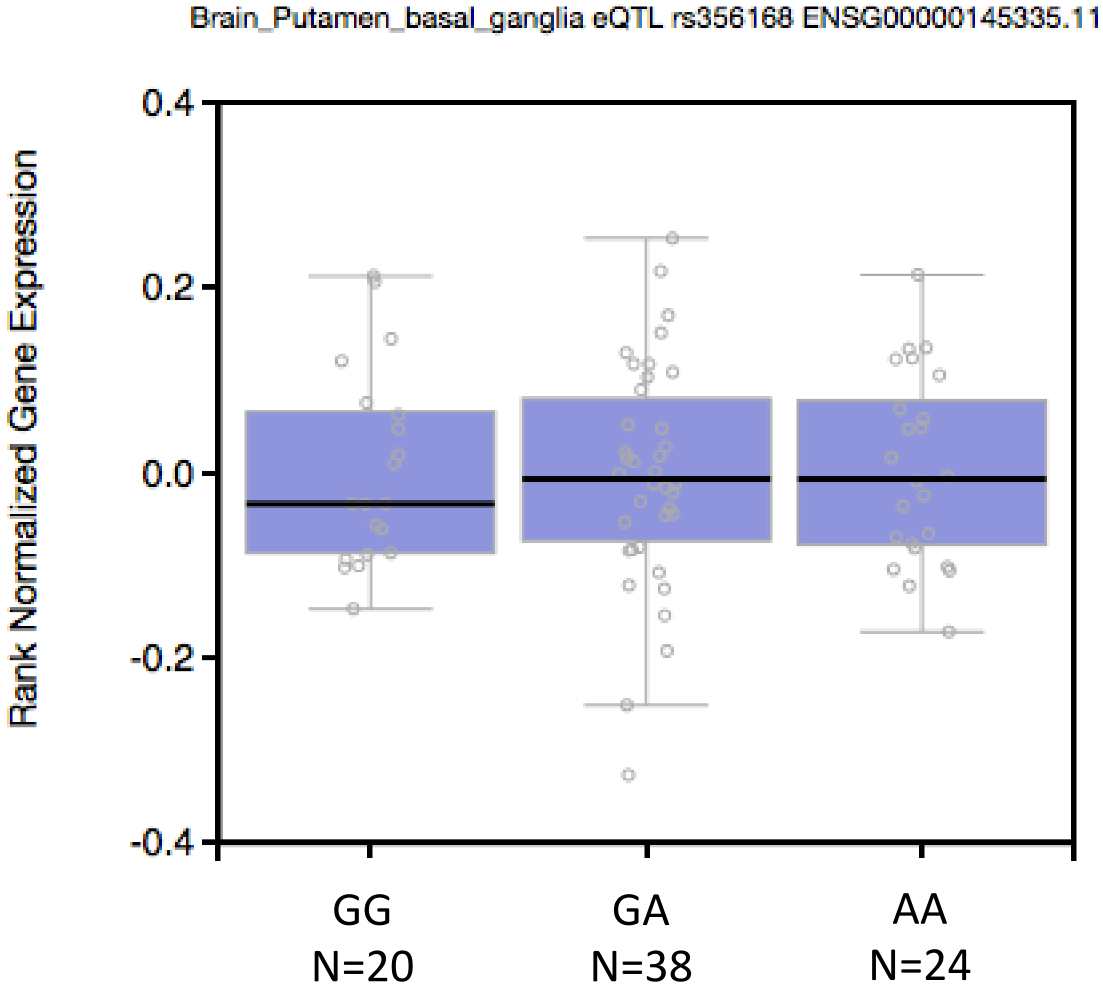

The Genotype---Tissue Expression (GTEx) Project was supported by the Common Fund of the Office of the Director of the NaYonal InsYtute of Health, and by NCI, NHGRI, NHLBI, NIDA, NIMH, and NINDS. The data used for the reported analyses were obtained from the GTEx Portal on 05/02/17.
